# Guinea pigs: A suitable animal model to study lipoprotein metabolism, atherosclerosis and inflammation

**DOI:** 10.1186/1743-7075-3-17

**Published:** 2006-03-27

**Authors:** Maria Luz Fernandez, Jeff S Volek

**Affiliations:** 1 Department of Nutritional Sciences University of Connecticut, Storrs, CT 06269, USA; 2 Department of Kinesiology University of Connecticut, Storrs CT 06269, USA

## Abstract

Numerous animal models have been used to study diet effects on cholesterol and lipoprotein metabolism. However, most of those models differ from humans in the plasma distribution of cholesterol and in the processing of lipoproteins in the plasma compartment. Although transgenic or knock-out mice have been used to study a specific pathway involved in cholesterol metabolism, these data are of limited use because other metabolic pathways and responses to interventions may differ from the human condition.

Carbohydrate restricted diets have been shown to reduce plasma triglycerides, increase HDL cholesterol and promote the formation of larger, less atherogenic LDL. However, the mechanisms behind these responses and the relation to atherosclerotic events in the aorta have not been explored in detail due to the lack of an appropriate animal model. Guinea pigs carry the majority of the cholesterol in LDL and possess cholesterol ester transfer protein and lipoprotein lipase activities, which results in reverse cholesterol transport and delipidation cascades equivalent to the human situation. Further, carbohydrate restriction has been shown to alter the distribution of LDL subfractions, to decrease cholesterol accumulation in aortas and to decrease aortic cytokine expression. It is the purpose of this review to discuss the use of guinea pigs as useful models to evaluate diet effects on lipoprotein metabolism, atherosclerosis and inflammation with an emphasis on carbohydrate restricted diets.

## Background

The use of appropriate animal models to determine the effects of dietary interventions on metabolic process and gene expression regulating cholesterol and lipoprotein metabolism is essential to understand the mechanisms underlying the reported effects on plasma lipids. In our previous reviews, we have shown the suitability of guinea pigs to study alterations on cholesterol and lipoprotein metabolism induced by diet [1] and by drug treatment [2]. More recent studies in this animal model evaluating diet-induced atherosclerosis [3,4], and dietary effects on alterations in the morphology and concentration of specific lipoprotein subfractions [5] add support to the appropriateness of this model.

**Figure 1:**
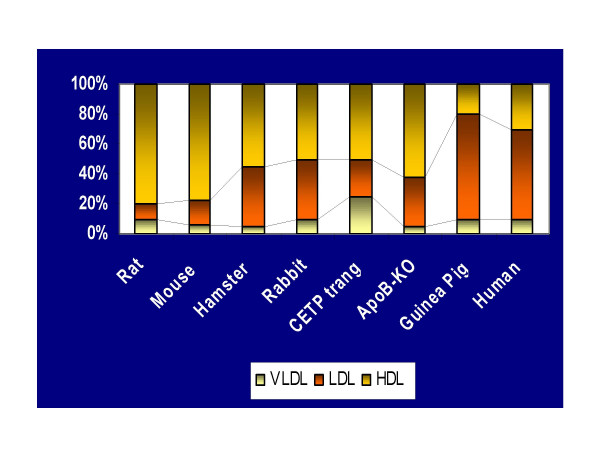
Comparisons between Lipoprotein Cholesterol Distribution between several animal models and humans.

The most striking similarity between guinea pigs and humans is that the majority of circulating cholesterol is transported in LDL [6]. Other rodents present major differences in lipoprotein cholesterol distribution and even genetic manipulations result in dissimilar lipoprotein profiles when compared to humans (Figure 1).

Other key points supporting the use of guinea pigs as models for human cholesterol and lipoprotein metabolism include: **1**. Higher concentrations of free compared to esterified cholesterol in the liver [7] as shown in reports from clinical studies [8]. **2**. In contrast to other rodents [9], guinea pigs possess plasma cholesteryl ester transfer protein (CETP) activity [10], a critical component for human reverse cholesterol transport [11]. **3**. Guinea pigs also have lecithin:cholesterol acyltransferase (LCAT) [12], and lipoprotein lipase (LPL) [13] activities that contribute to remodeling of plasma lipoproteins, which results in the formation of lipoprotein subclasses with different morphologies and physiologic functions including their role in lipid accumulation in the aorta and development of the atherosclerotic plaque; **4**. Comparable to humans [14,15], guinea pigs exhibit moderate rates of hepatic cholesterol synthesis [16] and catabolism [17]. **5**. Similar to humans, the binding domain for the LDL receptor differentiates between normal and familial binding defective apo B-100 [18]. **6**. Apo B mRNA editing in liver is present in negligible amounts (< 1%) compared to 18 to 70% in other species [19]; **7**. Guinea pigs require dietary vitamin C [20], an important anti-oxidant that may play a role in oxidation and atherosclerosis. **8**. Females have higher HDL levels than males and ovariectomized guinea pigs have a plasma lipid profile similar to post-menopausal women [21]. **9**. In response to exercise, guinea pigs lower plasma triglycerides (TG) and increase plasma HDL cholesterol (HDL-C) [22]. **10**. Guinea pigs respond to dietary fat saturation [23], dietary cholesterol [24] and dietary fiber [25] by alterations in LDL cholesterol. **11**. In contrast to hamsters they do not possess a fore-stomach, which ferments fiber before reaching the small intestine [26]. **12**. It has also been shown that guinea pigs are good models for studying the mechanisms by which statins [27], cholestyramine [28], apical sodium bile acid transport (ASBT) inhibitors [29] lower plasma LDL cholesterol. More recently, we have demonstrated that guinea pigs can also be used to study the mechanisms by which certain drugs affect triglyceride metabolism [30,31]

### Guinea pigs and dietary interventions

#### Dietary fat saturation

The clarification of some of the mechanisms by which dietary fatty acids alter plasma cholesterol concentrations and lipoprotein metabolism comes from the use of animal models [32,33]. Guinea pigs have been used as models to elucidate the mechanisms by which dietary fat saturation influences plasma lipids. They have lower plasma LDL cholesterol when the diet is rich in polyunsaturated fatty acids (PUFA) compared to saturated fat (SFA) intake [34]. We demonstrated that plasma cholesterol lowering was due to the up-regulation of the LDL receptor by PUFA and decreased conversion of VLDL to LDL [23]. In addition, we evaluated fatty acid chain length on hepatic cholesterol and lipoprotein metabolism. Stearic acid (18C) intake resulted in lower plasma cholesterol concentrations, palmitic acid (16) in an intermediate value and a diet rich in lauric (12C) and myristic (14C) acids had the greatest hypercholesterolemic effect [35]. The hypercholesterolemic effects of lauric/myristic were due to increased production/formation of VLDL associated with higher hepatic acyl CoA cholesteryl:acyltransferase (ACAT) activity [36] and to decreased plasma LDL turnover [35] associated with a lower number of LDL receptors determined in hepatic membranes calculated by Maximal binding (Bmax) [34].

#### Dietary fiber

Many studies have been conducted in guinea pigs to understand the secondary mechanisms by which dietary fiber lowers plasma LDL cholesterol [17,37,38]. Soluble fiber disrupts the enterohepatic circulation of bile acids by interfering with micelle formation in the intestinal lumen and increasing bile acid output. Decreases in cholesterol absorption have also been observed with intake of pectin (a source of soluble fiber) [25]. Since the recycling of bile acids is tightly regulated, the liver needs to synthesize more bile acids via hepatic cholesterol resulting in the up-regulation of cholesterol 7α-hydroxylase (CYP7), the regulatory enzyme of bile acid synthesis. As a result of the decrease in hepatic cholesterol, HMG-CoA reductase, the rate limiting and regulatory enzyme for cholesterol biosynthesis is up-regulated [17,25] and in addition, the LDL receptor is up-regulated to remove cholesterol from circulation [25]. These series of events result in the lowering of LDL cholesterol. Other mechanisms contributing to the lowering of LDL-C by fiber intake include decreases in ACAT activity [17,35], leading to a formation of a cholesteryl ester depleted VLDL, which does not get converted to LDL but is rather promptly removed from circulation by the LDL receptor [39].

#### Dietary cholesterol

Similar to humans [40], guinea pigs experience different responses to dietary cholesterol by which we could classify them as hyper- or hypo-responders [41]. Increasing dietary cholesterol results in accumulation of hepatic cholesterol and increased plasma cholesterol concentrations. One of the first mechanisms by which guinea pigs handle the excess of hepatic cholesterol is by suppressing HMG-CoA reductase activity. Decreases in LDL receptor in hepatic membranes has also been observed.

### Guinea pig and drug treatments

#### Reductase inhibitors

Guinea pigs are good models for the study of HMG-CoA reductase inhibitors. They experience significant decreases in LDL cholesterol even at the lowest doses [27]. Atorvastatin, a well known HMG-CoA reductase inhibitor lowers LDL cholesterol by decreasing apo B secretion from the liver, which leads to less conversion of VLDL to LDL [27]. In addition LDL size is significantly modified by atorvastatin. Increases in *in vivo *LDL turnover due to atorvastatin and simvastatin treatment has also been observed [42]. The mechanisms by which lovastatin lowers LDL-C were evaluated in guinea pigs [43] and found to be similar to a later report in humans [44]

#### Other drugs affecting LDL metabolism

Guinea pigs have also been shown to decrease LDL-C with apical sodium bile acid transporters (ASBT) inhibitors by interrupting the recycling of bile acids and increasing fecal bile acid ouptut [45]. Cholestyramine has also been shown to significantly reduce LDL-C in guinea pigs [28,46]. Specific changes in LDL particle associated with faster LDL catabolic rate have been proposed as one of the mechanisms by which cholestyramine exerts its hypocholesterolemic effect [44]. Other drugs such as microsomal transfer protein (MTP) inhibitors [31] have also been shown to decrease plasma LDL-C.

#### Drugs affecting triglyceride metabolism

Recently we have shown that guinea pigs also respond to drugs known to affect TG metabolism in humans. One of them is the MTP inhibitor, which was shown to reduce plasma TG without increasing hepatic lipid accumulation. [30] Also, rapamycin, a mammalian target of rapamycin (mTOR) inhibitor, prescribed to organ transplant patients is known to induce hypertriglyceridemia. We observed significant increases in plasma TG in guinea pigs treated with low and moderate doses of rapamyin for 3 wk.[31]

### Models for atherosclerosis and inflammation

In addition to the responses on plasma lipids due to a dietary intervention or drug treatment, a suitable animal model should be able to develop atherosclerosis, the ultimate outcome of extended hypercholesterolemia or from circulating atherogenic lipoproteins. We have shown that guinea pigs develop atherosclerosis and that gender and hormonal status affect the extent of the atherosclerotic plaque [47]. More recently we have shown that high cholesterol diets induce aortic cholesterol accumulation and that certain dietary components or drug treatment can reduce concentrations of cholesterol in the aorta even in the presence of very high dietary cholesterol [43,48].

**Figure 2:**
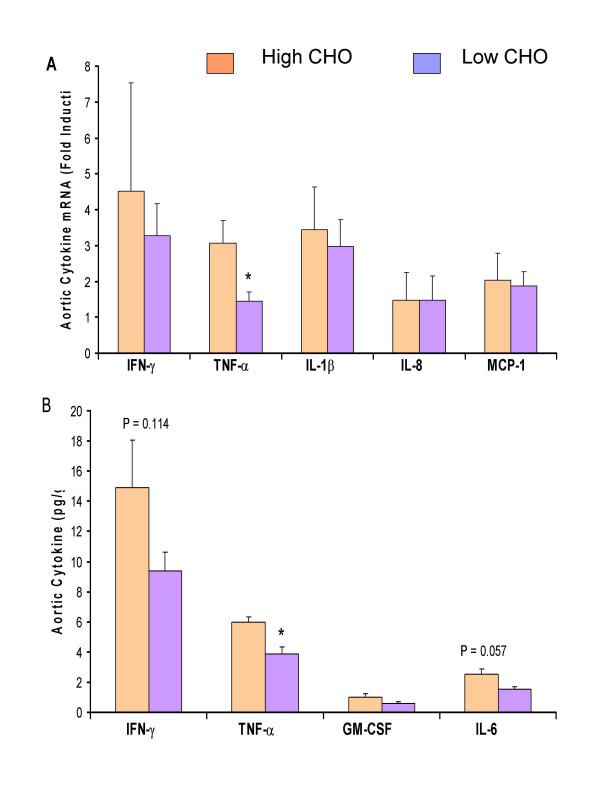
Gene expression (A) and Protein levels (B) of several inflammation markers in aortic tissues of guinea pigs (mean ± SEM, n = 10 guinea pigs per group) fed either a low (12% energy) or a high (40% energy) carbohydrate containing 0.25% cholesterol.

Atherosclerosis is no longer simply viewed as a disease of cholesterol accumulation in the arterial wall, but rather as a process that involves low-grade vascular inflammation in all stages. Given the critical nature of inflammation, an ideal animal model of atherosclerosis should resemble the human disease both in terms of cholesterol metabolism and the inflammatory process. We have validated the guinea pig as a model to study the inflammatory component of diet-induced atherosclerosis. We measured proinflammatory cytokine protein and mRNA expression in the aortas of guinea pigs fed high-cholesterol diets either high or low in carbohydrate for 12 wk. We observed a significant increase in aortic cytokines in guinea pigs fed high cholesterol compared to low cholesterol diets (unpublished results). When we compared aortic protein concentrations of interferon (IFN)-γ, tumor necrosis factor (TNF)-α, and interleukin 6 (IL-6), we found that carbohydrate restriction decreased TNF-α gene expression (Fig 2A) and protein (Fig 2B). We used real-time primers for guinea pig IFN-γ, TNF-α, IL-1β, IL-8, and MCP-1 and quantitative real-time PCR. Thus, we clearly showed an atherogenic inflammatory process in guinea pigs fed high-cholesterol as quantified by both protein and mRNA expression. We also demonstrated that macronutrient composition may alter inflammatory responses.

### Guinea pigs as models for the study of carbohydrate restricted diets

#### Lipoprotein subclasses

**Figure 3:**
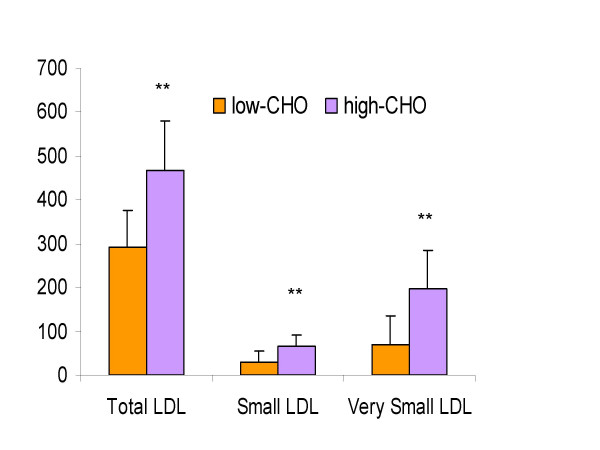
Concentration of plasma total number of LDL particles and smaller LDL subfractions guinea pigs (mean ± SEM, n = 10 guinea pigs per group) fed either a low (12% energy) or a high (40% energy) carbohydrate containing 0.25% cholesterol. ** significantly different (P < 0.001)

Carbohydrate restriction has been shown to alter triglyceride metabolism [49]. One major effect of carbohydrate restriction on lipoprotein metabolism is the formation of larger more buoyant LDL particles typical of pattern A the less atherogenic form [50]. Small dense LDL particles classified as pattern B are associated with a much higher risk for cardiovascular disease [51], thus this important feature of carbohydrate restriction in increasing the size of LDL is quite beneficial, specifically for patients with the metabolic syndrome and type II diabetes, which have a predominance of pattern B LDL particles. We have shown in guinea pigs that the distribution of LDL particles as measured by nuclear magnetic resonance shifts to larger LDL when guinea pigs are fed a low carbohydrate (12% energy) versus a high carbohydrate (40% energy) diet (52) (Figure 3).

#### Carbohydrate restriction and cytokines and aortic cholesterol accumulation

Because carbohydrate restriction decreases the number of small LDL in guinea pigs [52], we speculate that there is less penetration of LDL particles through the arterial wall. Because of the unregulated uptake of cholesterol by macrophages, foam cell formation is increased in the presence of oxidized LDL in the intima [53]. These foam cells are responsible for the activation of T cells and B cells, which promote the secretion of cytokines [54]. Thus it is not surprising to observe a decreased cytokine protein and mRNA abundance in aortas of guinea pigs fed a high versus a low carbohydrate diet (Fig. 2). Another important observation was that guinea pigs fed the CRD had less accumulation of cholesteryl ester in the aorta supporting our observation that the higher concentrations of smaller LDL (as produced by the high carbohydrate diet) may result in higher cholesterol accumulation in aorta. These findings suggest that carbohydrate restriction effects on plasma lipoproteins have a direct impact on inflammation and atherosclerosis in guinea pigs. We have studies underway to confirm these findings and provide additional insight into the mechanisms by which carbohydrate restriction affects lipoprotein and inflammatory aspects of atherosclerosis in guinea pigs.

## Conclusion

In this review, we have demonstrated that guinea pigs are excellent models to evaluate the mechanisms by which diet interventions and drug treatments alter plasma lipids and lipoprotein metabolism. Further, we have shown that guinea pigs have an inflammatory response and develop atherosclerosis when challenged with a high cholesterol diet and that diet treatment (as in the case of carbohydrate restriction) may prove to be beneficial in reducing the expression of inflammatory cytokines and atherosclerosis development. Further the guinea pig is proposed to evaluate in depth the metabolic alterations induced by CRD, which substantially improve plasma lipid profiles

## Abbreviations

ACAT: acyl CoA cholesteryl acyltransferase, Apo: apolipoprotein; B_max_: Maximal binding, ASBT: apical sodium bile acid transporters; CE: cholesteryl ester; CETP: cholesterol ester transfer protein; CETP transg: CETP transgenic;CRD: carbohydrate restricted diets; CYP7: Cholesterol 7α-hydroxylase; FC: free cholesterol; GM-CSF: granulocyte-macrophage colony stimulatory factor; HDL-C: HDL-cholesterol, HMG-CoA: 3-hydroxy-3-methylglutaryl coenzyme A; IL-6: interleukin 6, INF: interferon; LDL-C: LDL cholesterol; LCAT: lecithin-cholesterol acyltransferase; LPL: lipoprotein lipase; MONO: monounsaturated; mTOR: mamamalian target of rapamycin, MTP: microsomal transfer protein PUFA: polyunsaturated; SAT: saturated; TG: triglycerides; TNF-α: tumor necrosis factor alpha; VLDL-C: VLDL cholesterol

## Competing interests

The author(s) declare that they have no competing interests.

## Authors' contributions

Both MLF and JSV were responsible for review conception and design, interpretation, and critical revision of the manuscript.
